# Differential Cross Sections for Pair-Correlated Rotational Energy Transfer in NO(A^2^Σ^+^) + N_2_, CO, and O_2_: Signatures of Quenching Dynamics

**DOI:** 10.1021/acs.jpca.3c03606

**Published:** 2023-07-23

**Authors:** Thomas F. M. Luxford, Thomas R. Sharples, Martin Fournier, Clément Soulié, Martin J. Paterson, Kenneth G. McKendrick, Matthew L. Costen

**Affiliations:** Institute of Chemical Sciences, Heriot-Watt University, Edinburgh EH14 4AS, United Kingdom

## Abstract

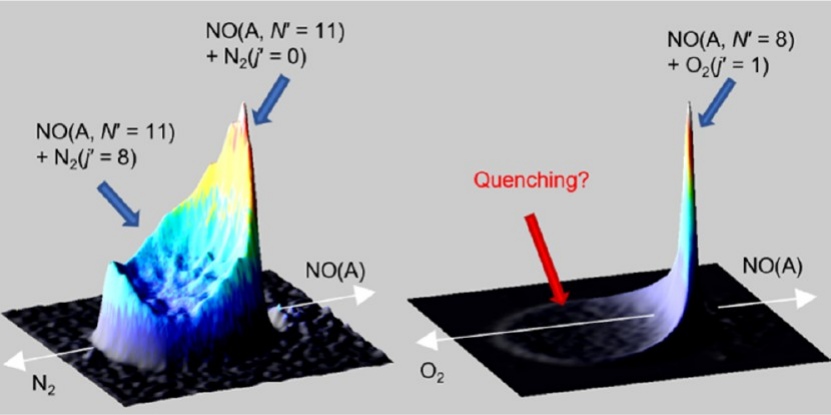

A crossed molecular beam, velocity-map ion-imaging apparatus has been used to determine differential cross sections (DCSs), as a function of collider final internal energy, for rotationally inelastic scattering of NO(A^2^Σ^+^, *v* = 0, *j* = 0.5*f*_1_) with N_2_, CO, and O_2_, at average collision energies close to 800 cm^–1^. DCSs are strongly forward scattered for all three colliders for all observed NO(A) final rotational states, *N*′. For collisions with N_2_ and CO, the fraction of NO(A) that is scattered sideways and backward increases with increasing *N*′, as does the internal rotational excitation of the colliders, with N_2_ having the highest internal excitation. In contrast, the DCSs for collisions with O_2_ are essentially only forward scattered, with little rotational excitation of the O_2_. The sideways and backward scattering expected from low-impact-parameter collisions, and the rotational excitation expected from the orientational dependence of published van der Waals potential energy surfaces (PESs), are absent in the observed NO(A) + O_2_ results. This is consistent with the removal of these short-range scattering trajectories via facile electronic quenching of NO(A) by O_2_, in agreement with the literature determination of the coupled NO-O_2_ PESs and the associated conical intersections. In contrast, collisions at high-impact parameter that predominately sample the attractive van der Waals minimum do not experience quenching and are inelastically forward scattered with low rotational excitation.

## Introduction

Experimental measurements of rotationally inelastic scattering provide direct information on the forces experienced in molecular interactions. The combination of crossed molecular beams and velocity-map ion imaging has been widely used to obtain state-to-state differential cross sections (DCSs), higher-order correlations involving initial and final rotational angular momenta, and the dependence of these observables on initial bond orientation.^1−8^ Combined with quantum and classical scattering calculations performed on accurate ab initio potential energy surfaces (PESs), these measurements have provided extensive insight into the roles of attractive and repulsive forces, interference effects, and resonance interactions in diatom–atom rotational energy transfer (RET).^9−16^

The NO radical, which combines open-shell character with experimentally convenient stability and accessible spectroscopy, has been the principal target of these investigations in its collisions with rare gas (Rg) atoms.^17,18^ NO has also proven particularly fruitful as an experimental species through the physical control that can be achieved on it using the Stark effect via the application of static electric fields.^19^ At the simplest level, this has enabled the selection of a single rotational state of NO(X^2^Π), prior to collision, from the initial rotational distribution within the molecular beam, thereby enabling true state-to-state measurements.^9,20^ The application of subsequent electric fields has allowed additional control. Brouard and co-workers have used a static quadrupole electric field to orient the bond axis of the selected state relative to the initial collision velocity, and have thereby measured state-to-state DCSs for N-end versus O-end collisions, as well as end-on versus side-on collisions, with a range of rare gas colliders. This work has demonstrated the importance of interference effects between collisions on the different ends and sides of NO in the final state-resolved DCSs.^6,13,14,21^

The Stark effect also provides a methodology for precise control of the velocity of an NO molecular beam, which has been exploited in a range of state-of-the-art experiments by van der Meerakker and co-workers. This precise velocity control provides very narrow collision energy distributions, which has allowed the resolution of diffraction oscillations in the inelastic scattering of NO(X) in collisions with He, Ne, and Ar.^10,12^ This methodology also enables deceleration of the NO to arbitrary velocities, which Meerakker and co-workers have used to perform precision experiments at collision energies down to ≈1 cm^–1^, and thereby to image the dynamics of scattering resonances, providing unprecedented insight into the PESs for these systems.^11,15,16^

An alternative approach to control the initial rotational quantum state is to use optical excitation. Suits and co-workers have demonstrated the use of stimulated-emission pumping to prepare single rotational quantum states of vibrationally excited NO for gas-phase inelastic collisions. By combining this with molecular beams with a small crossing angle and short pulses, they have demonstrated DCS measurements for low-energy collisions of NO(X, *v* = 10) with Ar and He, providing a method to test the accuracy of ab initio potentials in a new collisional regime.^22−24^

A further alternative, based on a different form of optical state preparation, is to perform scattering studies on electronically excited species. This provides an opportunity to study the dynamics of inelastic scattering with identical kinematics to that of the ground electronic state, but with a change in the PES. We have shown that NO(A^2^Σ^+^, *v* = 0, *j*) initial levels can be prepared in the scattering region of a crossed molecular beam apparatus, followed by state-specific detection of the rotationally inelastically scattered NO(A, *v* = 0, *N*′) products using velocity-map imaging, all within the ca. 200 ns fluorescence lifetime of NO(A).^25−28^ Using this methodology, we have extensively studied the dynamics of NO(A) + Rg collisions, determining both state-to-state DCSs and product polarization-dependent DCSs for collisions with He, Ne, Ar, and Kr.^29−31^ We have shown how the comparison of these detailed experimental measurements to close-coupled quantum scattering calculations may then be used as a sensitive test of the accuracy of the ab initio potentials.^32^

There have been substantially fewer crossed-beam VMI studies of molecule–molecule scattering at the same level of experimental and theoretical detail. This is fundamentally because of the additional level of complexity introduced by the presence of open product rotational energy channels in the collision partner. However, recently the techniques exploited so successfully on NO(X) + Rg collisions have begun to be applied to NO(X) + molecule systems. The precise velocity control enabled by Stark deceleration has particular benefits, enabling clear resolution of correlated rotation–rotation product channels with bimolecular colliders. Meerakker and co-workers have exploited this in NO(X) collisions with O_2_, CO, and D_2_/HD.^33−37^ In addition to providing stringent tests of scattering calculations on ab initio PESs, these measurements have also uncovered new inelastic scattering mechanisms, including the surprising observation of glory scattering in “hard” inelastic collisions in which substantial energy is transferred to rotation of the collision partner.^36,37^ In these experiments, the collider initial rotational distribution was defined purely by the cooling involved in the molecular beam expansion, and therefore although dominated by the lowest accessible rotational level, also contained small fractions of other rotational states. In principle, Stark or other state-preparation techniques can also be applied to the collider beam, allowing inelastic collisions that are fully state-to-state in both collision partners. This clearly presents a significant additional experimental challenge but has very recently been demonstrated by Meerakker and co-workers in the NO(X) + ND_3_ system, where the Stark-decelerated NO(X) collided with ND_3_ that was Stark hexapole state-selected.^38^

Although the high-precision velocity control enabled by Stark or Zeeman decelerators clearly provides great benefits, it is possible to determine information on the DCS correlated with the degree of rotational excitation in the collision partner in more conventional crossed-beam VMI experiments.^39,40^ Significant new mechanistic insight can still be provided by these lower-resolution experiments, as demonstrated by Sun et al., who uncovered a new mechanism in CO + CO inelastic scattering, in which both CO molecules experienced high rotational excitation while undergoing forward scattering, which they dubbed forward-scattered symmetric excitation (FSSE). Quasi-classical scattering calculations and comparison to CO + N_2_ inelastic scattering, in which FSSE scattering was not observed, revealed that this mechanism was mediated by the dipole–dipole interaction in CO + CO.^41^ Finally, we have also demonstrated angular scattering information as a function of (post-collision) collider internal energy for the NO(A, *v* = 0, *j* = 0.5) + N_2_ system, with the NO optically prepared.^42^ In this system, strong forward scattering was observed for low-*N*′ rotational states of NO in coincidence with low rotational excitation of N_2_. Although the majority of all NO *N*′ states were formed in coincidence with low rotational excitation of the N_2_, some moderate rotational excitation of the N_2_ was observed for higher-NO *N*′ states. This primarily correlated with sideways scattering, while forward and backward scattering of high-*N*′ NO was mostly observed in coincidence with low rotational excitation of N_2_. However, significant difficulties were found in the analysis of the images to determine the scattering distributions for this system, arising from the strong forward scattering observed, as a result of which we were unable to analyze the 0 to ≈15° scattering-angle range.

In this paper, we present new experimental results on rotationally inelastic collisions of NO(A, *v* = 0, *j* = 0.5) with the molecular colliders O_2_ and CO. While these colliders have similar masses, and hence similar collision kinematics, they have very different rate constants for electronic quenching of NO(A).^43^ At the collision energies used in our experiments, the quenching cross sections can be summarized as ≈0.3 Å^2^ for N_2_, ≈8 Å^2^ for CO, and ≈25 Å^2^ for O_2_. We have analyzed these new data, and also our previously published data for collisions with N_2_,^42^ with an improved approach that extracts the DCSs as a function of internal energy of the molecular collider, and is also capable of fully fitting the strongly forward scattered distributions observed in these systems. There are clear systematic differences in the degree of rotational excitation in both NO(A) and collision partner, and the correlated DCSs, for all three systems, despite their very similar kinematics and collision energies. In particular, very little rotational excitation is observed in either fragment for collisions with O_2_, for which the DCSs are also almost exclusively forward scattered. We discuss these results in the context of the substantially different electronic quenching kinetics of NO(A) with the three colliders, and recent experimental and theoretical work on the quenching dynamics and PESs of NO(A) + O_2_, N_2_, and CO.^44−48^

## Experimental Methods

The experimental apparatus has been described in detail before, and we only give an overview here.^28−31,42^ In brief, two pulsed molecular beams were crossed at right angles in the center of a stack of velocity-map ion-optics. A pulse of UV radiation at ≈226 nm, linearly polarized perpendicular to the molecular beam plane and resonant with the Q_1_(0.5) line of the NO(A^2^Σ^+^ – X^2^Π) (0,0) band, was used to excite NO molecules in one molecular beam to the NO(A, *v* = 0, *N* = 0, *j* = 0.5) state. Note that this rotational state cannot be aligned. The state-selected NO(A) then underwent collisions with N_2_, CO, or O_2_ from the second molecular beam. The products of rotational energy transfer to NO(A, *v* = 0, *N′*) were probed by a second laser pulse at ≈600 nm, resonant with the appropriate lines within the R-branch of the NO(E^2^Σ^+^ – A^2^Σ^+^) (0,0) transition. A delay of 370 ns between the preparation and probe laser pulses provided time for collisions but was short enough that none of the prepared NO(A) molecules could translate out of the observed preparation/probe volume. A third laser pulse at 532 nm ionized only the NO(E) molecules excited by the probe laser. The resulting NO^+^ ions were accelerated by the ion-optics and velocity-mapped onto a micro-channel plate detector with a phosphor screen, and captured with a CCD camera. The probe laser polarization was alternately set parallel to the plane of the molecular beams (horizontal, H) and at right angles (vertical, V), using a photoelastic modulator (Hinds, Inc. PEM-90).

10% NO (BOC, 99.998%) was seeded in Ne (BOC, 99.999%) with a backing pressure of 3 bar to produce a molecular beam with a speed distribution well described by a Gaussian with a mean of 815 m s^–1^ and full width at half-maximum (FWHM) of 57 m s^–1^. Pure beams of the molecular collision partners, N_2_, CO, and O_2_ (all sourced from BOC with purities of 99.999%) were generated from backing pressures of 5 bar. The resulting molecular beam speeds, which were also well described by Gaussian distributions, and the associated average collision energies are provided in Table 1.

**Table 1:** Mean and FWHM of Gaussian-Distributed Molecular Beam Speeds, and Associated Gaussian Collision Energy Distributions for Collisions of NO with the Three Molecular Colliders

collider	mean speed (m s^–1^)	speed FWHM (m s^–1^)	mean collision energy (cm^–1^)	energy FWHM (cm^–1^)
N_2_	800	74	790	92
CO	767	74	759	89
O_2_	759	72	803	93

For NO(A) + N_2_, images were recorded for the final rotational states *N′* = 3 and 5–11, for CO the range was *N′* = 3 and 5–10, while for O_2_ the range was restricted to *N′* = 3 and 5–8; product signal levels were found to be too low to acquire images for *N′* ≥ 9 for O_2_. For each final rotational level of each system, six sets of individual images were acquired, each set consisting of signal and background images for both the V and H polarizations of the probe laser. The background images were acquired with the collider molecular beam delayed by 1 ms relative to the NO molecular beam. Each individual image set was the result of 64,000 camera shots, i.e., 16,000 shots across each of the V and H, signal and background images, which resulted from five repeated scans over the Doppler profile of the probe NO(E-A) transition. Subsequent data analysis was performed simultaneously on the V and H background-subtracted signal images, as discussed in more detail in the following section.

## Image Analysis

We have fitted the experimental images to extract the differential cross sections (DCSs) as a function of the internal energy (*E*_int_) of the unobserved collision partner, representing rotational excitation of the N_2_, CO, or O_2_. We henceforth represent the final rotational level of the collider as *j*′, and label the probed rotational level of the NO as *N*′, to clearly distinguish rotation of the two collision products. We have previously published a detailed description of a fitting methodology for collisions with atomic colliders.^29−31^ In this approach, basis images representing either DCS or rotational angular momentum alignment functions (e.g., Legendre polynomials) were simulated using a Monte-Carlo integration over the independently determined experimental parameters, i.e., molecular beam speed distributions, velocity-map-imaging resolution, etc. This simulation assumed, of course, that the collision partner had no internal degrees of freedom, with the consequence that the spread of final speeds of the product NO(A) in the collision frame was entirely determined by the spread of collision energies. Under the assumption that the dependence of the images on the DCS and alignment functions were separable, an iterative fitting procedure was then used. Experimental images were fitted with a linear combination of basis images dependent on the DCS functions, simulated with an assumed alignment moment distribution, to determine the DCS. This DCS was then used to generate a set of alignment moment-dependent basis images that were fitted to the experimental data to determine the alignment moments. This new set of alignment moments were used in a redetermination of the DCS, with the cycle repeated until the DCS and alignment moments converged. We henceforth refer to this as the “atomic” fitting software.

We extended this atomic approach to fit images arising from NO(A) + N_2_, described in a previous publication.^42^ This generalized the atomic fitting software, including the generation of basis images in which different amounts of the collision energy were transferred into the unobserved rotational modes of the collision partner. The spread of collision energies in the experiments was found to limit the resolution of the collision partner rotational excitation, with unacceptable levels of crosstalk apparent between basis functions separated by ≤60 cm^–1^. Extensive testing using simulations based on the DCSs found in scattering with rare gas collision partners gave us confidence that this brute force approach could successfully extract DCSs as a function of collider rotational excitation for this system.

However, the experimental images for *N′* ≤ 10 from NO(A) + N_2_ scattering were found to include an extremely sharp forward-scattered feature, covering the 0–15° range. Fitting such a sharp feature with Legendre polynomial basis functions requires a large (>20) number of basis functions for each collision partner product internal energy, *E*_int_. We found that the fitting procedure became unstable under these conditions, and that fits which successfully reproduced the sharp forward feature contained unphysical oscillations in the sideways and backward scattering that were not present in the data. We were therefore unable to fit the entire angular scattering range with this approach and instead chose to exclude the extreme forward ≈0–10° range. The experimental images reported in this paper for collisions with O_2_ and CO are also dominated by extreme forward scattering. We hence cannot use the previously published fitting methodology reliably on these data.

**Figure 1:**
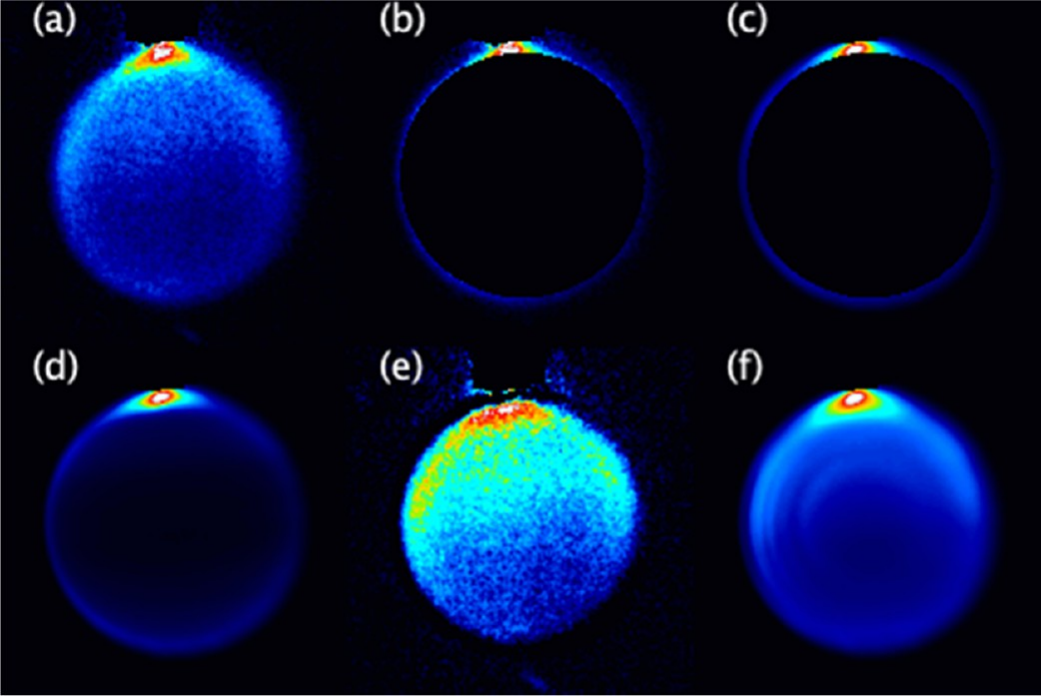
Images illustrating the fitting procedure, for NO(A) + N_2_, *N*′ = 10, H-polarization. (a) Full data image. (b) Data image after slicing for *E*_int_(1) in step 2. (c) Result of simultaneous fit to data in (b) and equivalent slice for V-polarization (not shown). (d) Full image reconstruction from fit shown in (c). (e) Data image resulting from the subtraction of (d) from (a), ready for slicing for *E*_int_(2). (f) Final complete fit image arising from 6 cycles of the slicing procedure.

We have therefore developed a new fitting methodology to overcome this limitation, which we describe here for the first time. This combines the basis function generation and fitting methods used in our previous programs with a variant of the “peeling” approach described by Brouard and co-workers.^39^ The software proceeds in the following fashion, as illustrated in Figure 1 for NO(A) + N_2_, *N′* = 10.

Step 1: Basis images, *I*_iso_(*n*), are generated, assuming an isotropic DCS and no rotational angular momentum polarization, for each of the *n* = 1, 2, ···, *n*_max_ collision partner product internal energies, *E*_int_(*n*), using the previously described Monte-Carlo procedure. Here *n* is an index, and in the results presented here *E*_int_(*n*) have been chosen as discrete rotational excitations of the product partner, starting with elastic scattering, i.e., Δ*j*′ = 0. As *E*_int_(*n*) increases with *n*, these images form a set of nested near circles of decreasing radius. A complete set of basis images, *I*_H/V_^Bas^(*n*, *l*_*n*_), representing different scattering-angle functions, *l*_*n*_, (in the work presented here these are evenly spaced triangles, but other functions including Legendre polynomials are program-selectable options) for each of the *E*_int_(*n*) was also generated at this time, including, if required, the effects of rotational angular momentum polarization from theoretical predictions.

Step 2: Pixels in the basis images generated for an isotropic DCS, *I*_iso_(*n*) and *I*_iso_(*n* + 1), are compared. Those pixels where *I*_iso_(*n* + 1) was less than a user-defined fraction (for the work here, 0.5) of *I*_iso_(*n*) are identified. These pixels form an outer slice, where the experiment is mostly only sensitive to the *E*_int_(*n*) product channel. Pixels are also identified where *I*_iso_(*n*) is less than a user-defined fraction (here, 1 × 10^–3^) of the maximum *I*_iso_(*n*). This is used to exclude areas of the image where the scattered signal size approaches the experimental background noise level, and thus where the experimental image has no information relevant to scattering for this *I*_iso_(*n*).

Step 3: For the pixels selected in step 2, a linear combination of the basis images, *I*_H/V_^Bas^(*n*, *l*_*n*_), was then fitted to the experimental images, *I*_H/V_^Bas^(*n*). The fitting was performed using a downhill simplex algorithm, with the constraint that the DCS remained positive, starting from an initially isotropic DCS. Multiple restarts of the simplex were performed to determine the global minimum. Figure 1a displays an initial complete experimental data image, in this case NO(A) + N_2_, *N*′ = 10, H-polarization. Figure 1b shows the image obtained for *E*_int_(1) = 0 cm^–1^ after the pixel selection procedure, with Figure 1c showing the result of the fit to the basis functions.

Step 4: The DCS determined from step 3 for *E*_int_(*n*) was now used to “peel” the experimental images. Complete images, covering all pixels in the original data, were simulated for *E*_int_(*n*) with the determined DCS. These simulated images were subtracted from the original experimental images, to leave new experimental images from which the contribution of *E*_int_(*n*) products had been removed. Figure 1d shows the full image simulated with the DCS determined in Figure 1c, and Figure 1e shows the result of the peeling subtraction of this simulation from the image in Figure 1a.

Step 5: We now returned to step 2, incrementing *n* by 1, and using the new “peeled” experimental images generated in step 4. This loop was repeated until *n* = *n*_max_, where the remaining pixels at the center of the image were fitted. Figure 1f shows the final result of the fitting procedure, summing the independent fits to each *E*_int_(*n*) for comparison to the data in Figure 1a.

In order to test the fitting code described above, we refitted some data for NO(A) + Ne scattering at an average collision energy of 523 cm^–1^. A complete analysis of these data using our atomic-collider fitting software has been published previously, here we compare the results of fitting these data using the peeling software with those previous results.^29^ Since Ne is a structureless collision partner, a perfect analysis of these data by the peeling software would result in a nonzero DCS for only the elastic channel, *E*_int_(1) = 0 cm^–1^, that also agreed with our previously published NO(A) + Ne DCSs.

**Figure 2:**
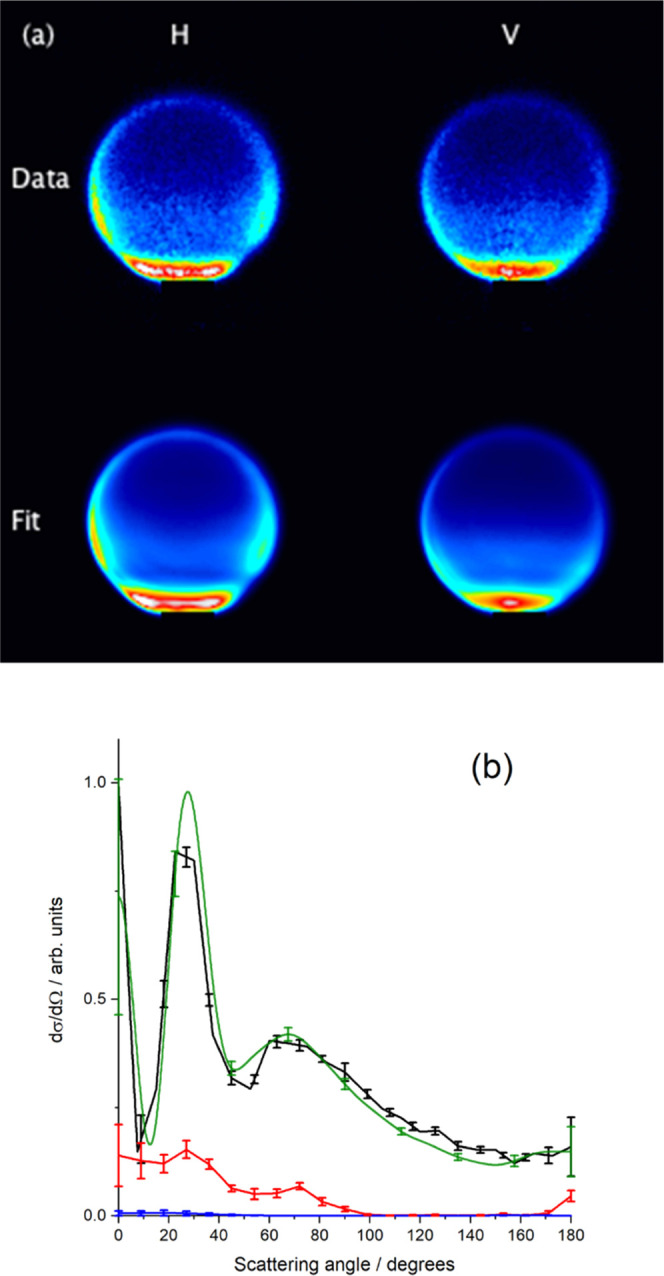
(a) Experimental data and fit images for NO(A) + Ne, *N*′ = 7, for both horizontal and vertical probe laser polarizations. (b) Differential cross sections resulting from the fit in (a), for *E*_int_(1) (black), *E*_int_(2) (red), and *E*_int_(3) (blue), compared to that previously reported from the atomic fitting procedure (green).^29^

The experimental details used in MC basis function generation in the new analysis software were identical to those used in our previously published analysis. Basis functions were generated with the assumption of 5 different collider final internal energies, based on those used in the analysis of data acquired from scattering with N_2_ (vide infra), namely, 0, 84, 144, 220, and 312 cm^–1^. The angular distributions have been described by evenly spaced triangular functions, as introduced in our recent study of NO(A) + Kr stereodynamics.^28^ These functions are well suited to the fitting of strongly forward scattered data and have been used in fitting the data from collisions with N_2_, O_2_, and CO presented in this paper. The scattering of NO(A) by Ne results in strong angle-dependent rotational alignment moments, which significantly affect the relative intensity of the images acquired with H or V laser polarizations. Our previous work has shown that quantum scattering calculations successfully reproduce the experimentally measured alignment moments, and hence the basis images were generated assuming those previously predicted alignment moments.^29^Figure 2a shows the H and V experimental images for scattering of NO(A) with Ne to product state *N*′ = 7, together with the fitted images produced by the new peeling software. For both experiment and fit, the V and H images are the sum of 8 independent experimental measurements, which were themselves fitted independently. There is excellent agreement between the experimental data and the fitted images. Figure 2b shows the resulting differential cross sections for the different *E*_int_(*n*), together with the DCS from fitting the same data using the “atomic” fit previously reported.^29^ There is an excellent, effectively quantitative agreement between the reported DCS for *E*_int_(1) and that found by the “atomic” fit. The peeling fit reports a small predominately forward DCS for *E*_int_(2), which has an integral cross section 11% of that for *E*_int_(1). The integral cross sections for *E*_int_(*n >* 2) are less than 0.2% in all cases. These results demonstrate that the peel fitting approach can accurately determine a DCS for a specific *E*_int_(*n*) with only modest crosstalk from other *E*_int_(*n*).

**Figure 3:**
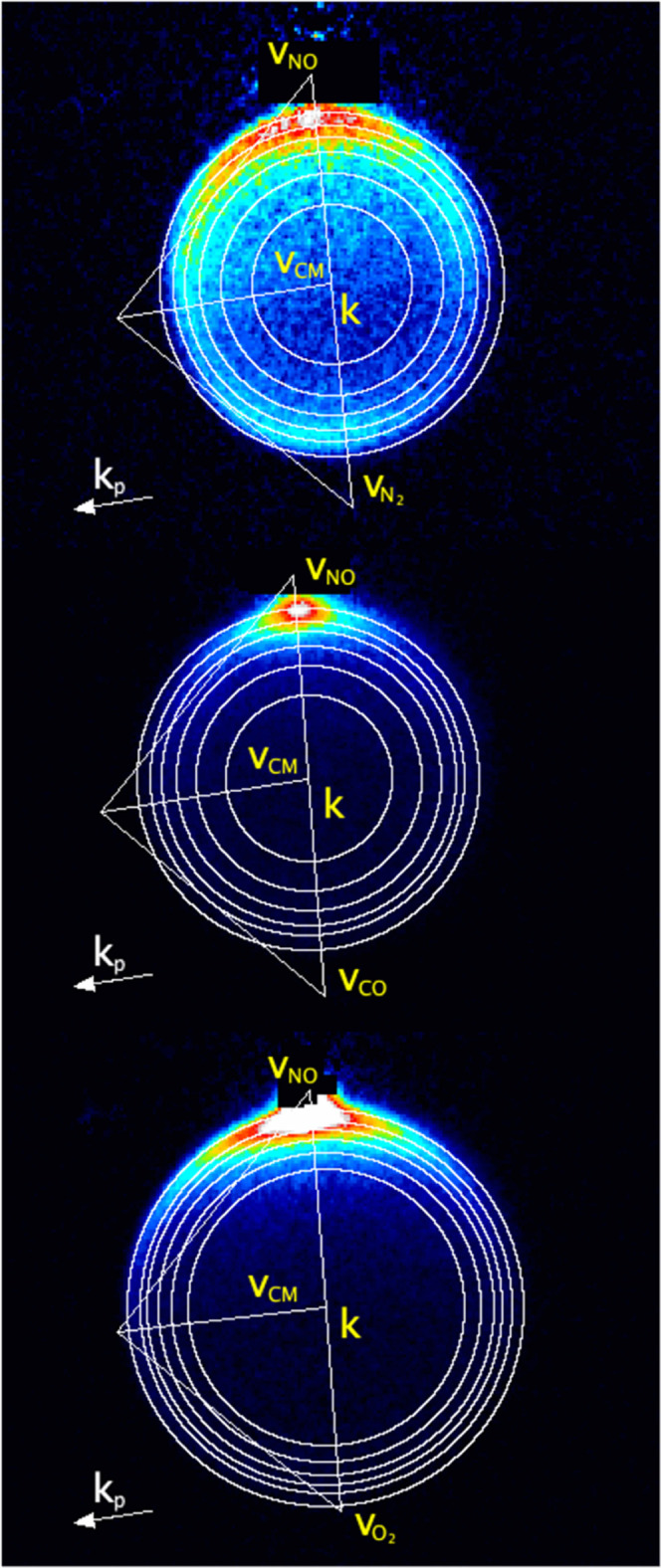
Newton diagrams for the three scattering systems, superimposed on experimental data images. Top: NO(A) + N_2_ for product level *N*′ = 11. Middle: NO(A) + CO for product level *N*′ = 10. Bottom: NO(A) + O_2_ for product level *N*′ = 8, with maximum intensity set to 20% of peak signal. Labels indicate the average laboratory frame velocities of: NO, **v**_NO_; relevant collider N_2_, CO, or O_2_, **v**_N2/CO/O2_; relative collision velocity, **k**; and velocity of the center of mass, **v**_CM_. Also indicated is the propagation direction of the probe laser, **k**_p_.

We used this peeling methodology to fit the experimental data from collisions with O_2_ and CO, which is presented here for the first time, and to fit the previously reported N_2_ data over the entire angular range for direct comparison. As noted above, triangular basis functions spaced by 5° were used to represent the DCS, as these localized basis functions have been demonstrated to be well suited for the fitting of images with strong forward scattering.^28^ For each collider, 6 final internal energies were included, reported in Table 2. In each case, *E*_int_(1) = 0 cm^–1^ represents elastic scattering, and scattering into a range of low-*j*′ levels (Δ*j*′ ≤ 6). Our experimental collision energy resolution is not high enough to separately resolve these closely spaced product levels. We have chosen the subsequent energies to provide an energy spacing of ≈60 to 80 cm^–1^, comparable to the FWHM of the collision energy distribution, and therefore our expected energy resolution. The specific energies chosen correspond to transfer from initial level *j* = 0 (N_2_ and CO) or *j* = 1 (O_2_) to different specific final rotational levels. Note that we therefore do not attempt to resolve all possible product channels, and we do not consider the initial rotational-state distributions of the colliders. The corresponding in-plane final scattering velocities are shown on Newton diagrams in Figure 3, superimposed on example scattering data for each of the colliders. Inspection of the experimental images for H and V geometries reveals differing intensities as a function of both DCS angle and azimuthal projection angle, which indicates significant angle-dependent product rotational angular momentum polarization. We have modeled this using kinematic apse (KA) conservation, widely used in previous studies of inelastic scattering.^3,9,10,20^ The scattering-angle-dependent angular momentum moments, *A*_0_^(2)^(θ), *A*_1+_^(2)^(θ), and *A*_2+_^(2)^(θ), were calculated for each *E*_int_(*n*) separately, and the corresponding *I*_H/V_^Bas^(*n*, *l*_*n*_) were simulated including the relevant probe laser sensitivities to these moments.

**Table 2:** Internal Energies, *E*_int_(*n*), together with Equivalent Final *j*′, Used in the Image Fitting for the Three Colliders N_2_, CO, and O_2_

N_2_		CO		O_2_	
*j′*	*E*_int_ (cm^–1^)	*j′*	*E*_int_ (cm^–1^)	*j′*	*E*_int_ (cm^–1^)
0	0	0	0	1	0
6	84	6	81	7	78
8	144	8	139	9	127
10	220	10	212	11	187
12	312	12	301	13	259
14	420	14	405	15	342

## Results

**Figure 4:**
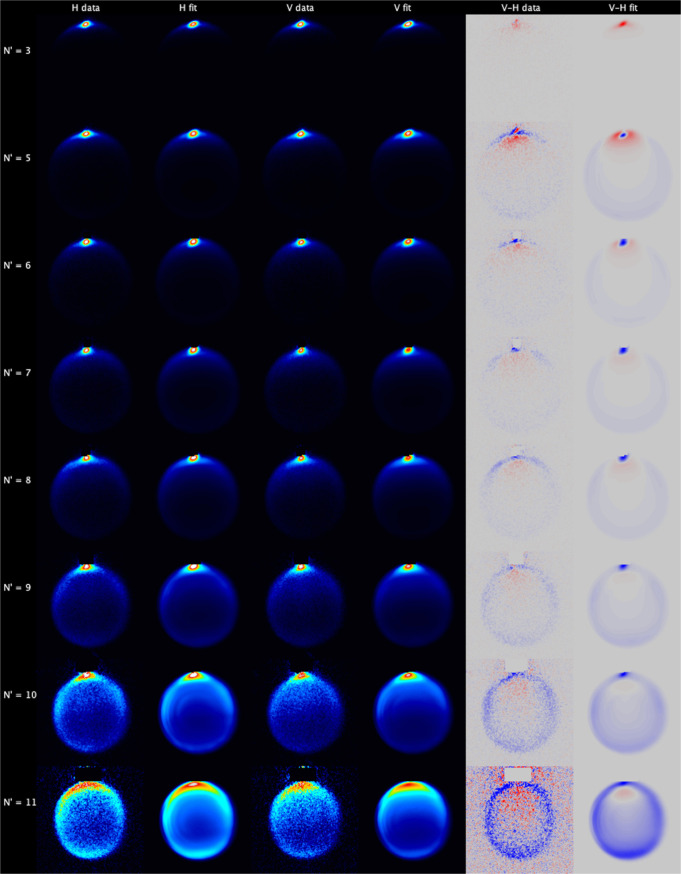
Experimental data and fits for NO(A, *j* = 0.5) + N_2_. Left to right: H-polarization data; H-polarization fit; V-polarization data; V-polarization fit; V–H data subtraction; V–H fit subtraction. Top to bottom: *N*′ = 3, 5, 6, 7, 8, 9, 10, and 11. The V–H subtraction color map ranges from blue (negative) to red (positive). The 6 experimental measurements for each *N*′ have been fitted independently, and the relevant images are summed for presentation here.

Figure 4 shows the data and fit images for NO(A, *j* = 0.5) collisions with N_2_, for both H and V geometries, as well as the V–H subtractions, which illustrate the effects of product rotational alignment. In each case, the image is the sum of the 6 independent experimental measurements, which were fitted independently. The data (but not the improved versions of the fits) have been reported previously, but we describe them again here in order to contrast them with the new CO and O_2_ data.^42^ The data images for *N*′ ≤ 10 all display a very sharp forward scattering peak centered on 0°. We emphasize that this is not an artifact resulting from incomplete subtraction of a beamspot. Beamspots are present in our experiment, arising from 2-photon 532 nm nonresonant ionization of the NO(A, *j* = 0.5) initial level. For *N*′ ≥ 7, the beamspot lies outside the scattering ring and is clearly visible as a masked region in the V–H data images. For lower *N*′, the ionization fluence was adjusted to ensure that the beamspot signal was significantly smaller (<1/3) than the resonant scattering signal, ensuring successful subtraction of the beamspot, which has a clearly different shape to the forward scattering signal.

For *N*′ = 3, nearly all scattering intensity is in this forward-scattered feature, and there is no evidence of “in-filling” of the scattering ring consistent with rotational excitation of the N_2_. As expected from classical models of linear to angular momentum transfer, as *N*′ increases the scattering intensity increases in the sideways and, eventually, backward directions. For *N*′ ≥ 9, the images are clearly noncircular, instead displaying a broadly oval shape with the major axis running along **k**. This is an indication of preferential energy transfer to the N_2_ for sideways scattering, resulting in lower scattering speeds for sideways scattered NO in these states. The V–H subtractions show that for most states and scattering angles, the H image has higher intensity. This is consistent with the product rotational angular momentum vector preferentially lying perpendicular to **k**. The fits, presented here for the first time using the newly developed peeling code, describe the data very well, with no systematic disagreement between data and fit for any of the rotational levels. There is also a good overall agreement between the V–H data and fit images, particularly at higher *N*′, implying that the KA model is a good approximation for the NO product angular momentum polarization.

**Figure 5:**
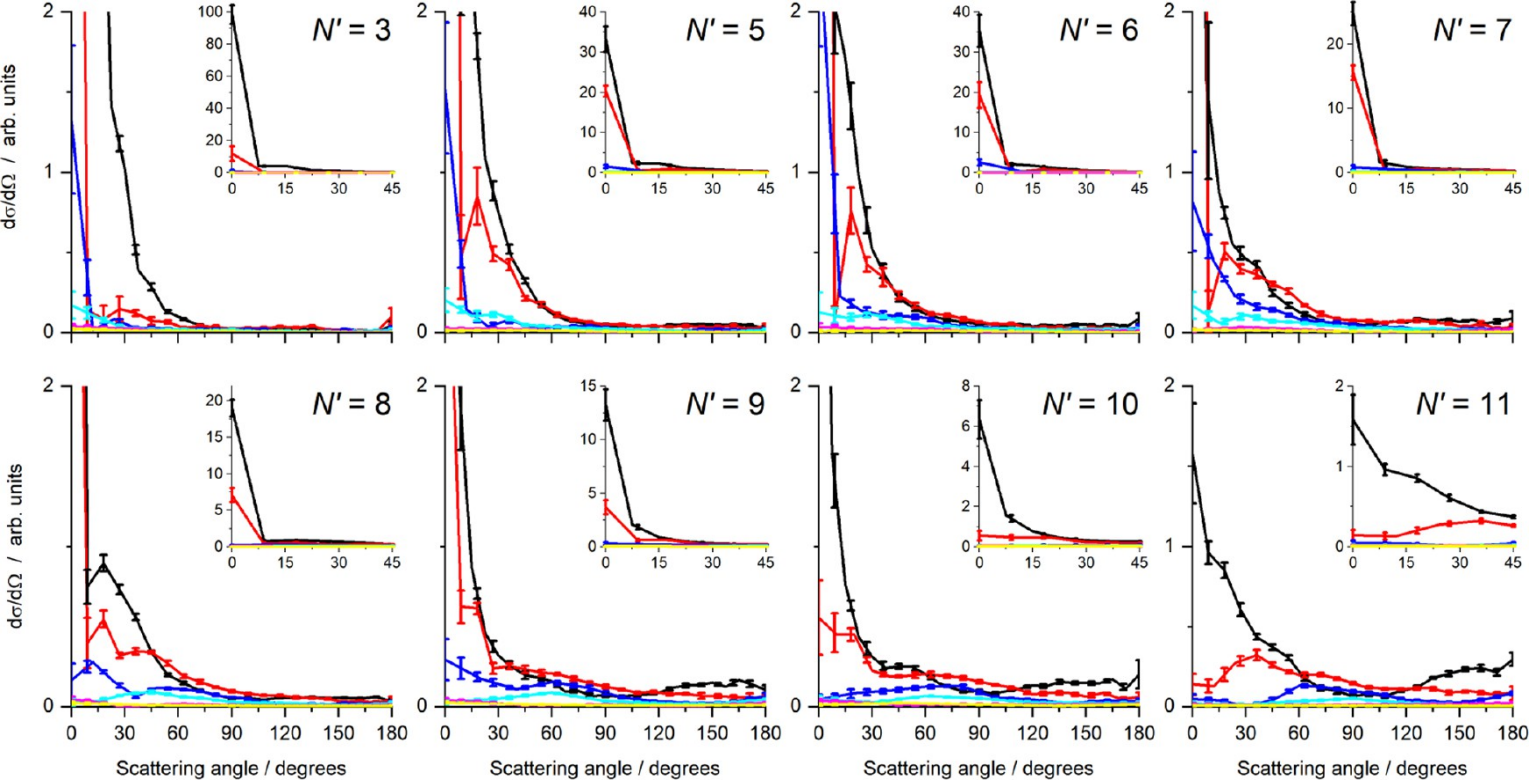
Differential cross sections (DCSs) for collision with N_2_ as a function of N_2_ internal energy and for final NO, *N′* = 3, 5–11. The total integral cross section for each final state has been normalized to unity. The main graphs span the full angular range (0–180°) but a reduced DCS range to enable comparison of DCSs for different N_2_ internal energies. The insets cover a limited angular range (0–45°) and the full DCS range, to enable comparison of the extreme forward scattering. Color scheme: *E*_int_(1) (black); *E*_int_(2) (red); *E*_int_(3) (blue); *E*_int_(4) (cyan); *E*_int_(5) (magenta); *E*_int_(6) (yellow). In each case, the error bars represent 1 standard error of the mean, resulting from the 6 independent experimental measurements of each *N*′.

Figure 5 shows the mean DCSs determined from the fits shown in Figure 4, as a function of internal energy in the unobserved N_2_, with error bars representing 1 standard error in the mean from the 6 independent measurements. As expected from the inspection of the data, they show very strong forward scattering, with the overall DCS for all final states peaking at 0°, together with increasing sideways and backward scattering as *N*′ increases. The relative magnitude of the DCSs for higher N_2_ energies gradually increases with increasing *N*′, indicating a positive correlation between *N*′ and *j*′. For *N*′ = 8 to 11, there is a growth in sideways scattering, correlated with rotational excitation of the N_2_ with *E*_int_(1) to *E*_int_(4). Little or no scattering is observed for the higher internal energies, *E*_int_(5) and *E*_int_(6). In contrast, the backward scattering is dominated by the *E*_int_(1) channel, for all *N*′.

**Figure 6:**
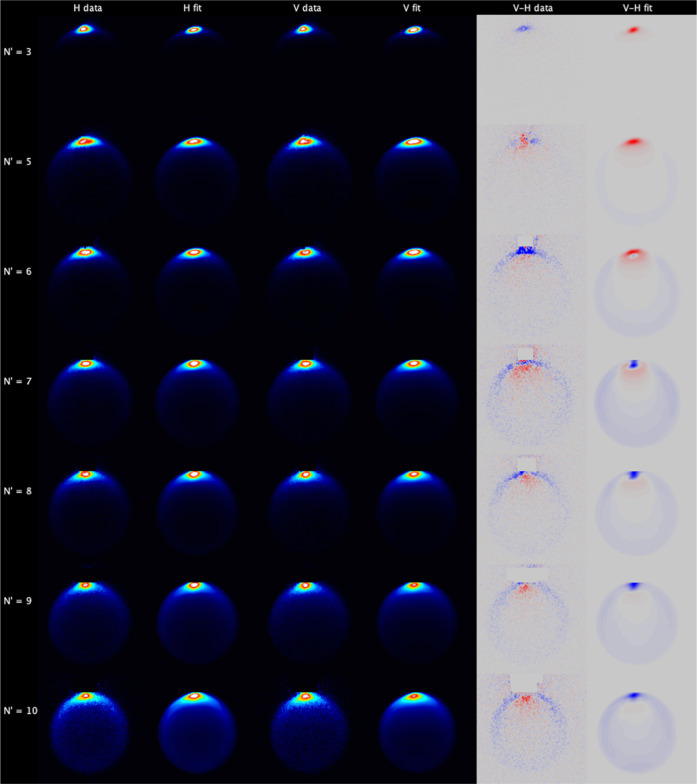
Experimental data and fits for NO(A, *j* = 0.5) + CO. Left to right: H-polarization data; H-polarization fit; V-polarization data; V-polarization fit; V–H data subtraction; V–H fit subtraction. Top to bottom: *N*′ = 3, 5, 6, 7, 8, 9, and 10. The V–H subtraction color map ranges from blue (negative) to red (positive). The 6 experimental measurements for each *N*′ have been fitted independently, and the relevant images are summed for presentation here.

**Figure 7:**
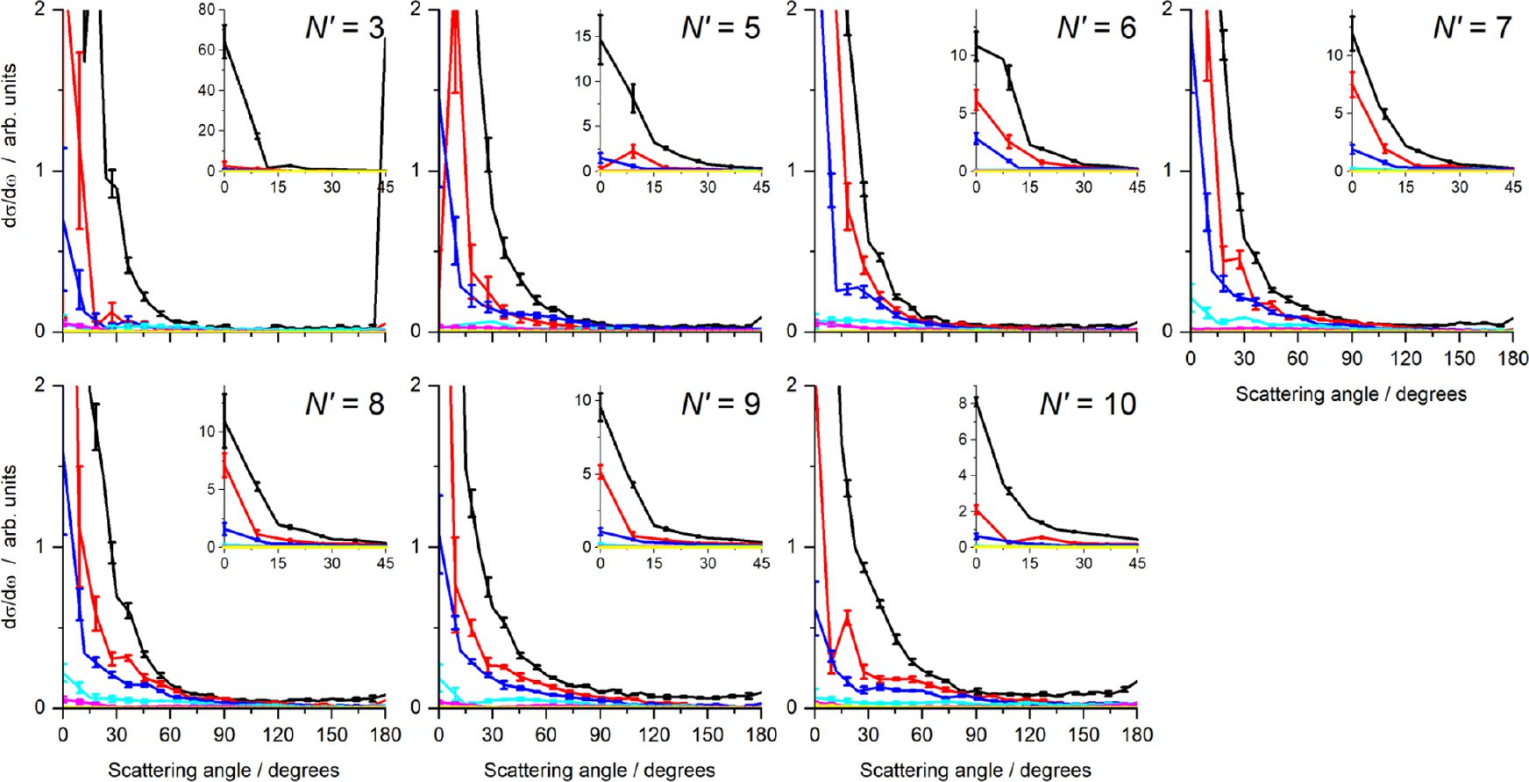
Differential cross sections (DCSs) for collisions with CO, as a function of CO internal energy and for final NO *N′* = 3, 5–10. The total integral cross section for each final state has been normalized to unity. The main graphs span the full angular range (0–180°) but a reduced DCS range to enable comparison of DCSs for different CO internal energies. The insets cover a limited angular range (0–45°) and the full DCS range, to enable comparison of the extreme forward scattering. Color scheme: *E*_int_(1) (black); *E*_int_(2) (red); *E*_int_(3) (blue); *E*_int_(4) (cyan); *E*_int_(5) (magenta); *E*_int_(6) (yellow). In each case, the error bars represent 1 standard error of the mean, resulting from the 6 independent experimental measurements of each *N*′.

Figure 6 shows the data and fit images for NO(A, *j* = 0.5) collisions with CO. At first inspection, they share many similarities to the N_2_ data. For all *N*′, the maximum intensity is observed for forward scattering, centered on 0°. The range of scattering angles increases as expected with increasing *N*′, although there is less visible sideways and backward scattering at the highest *N*′ than is observed for collisions with N_2_. Close inspection reveals that the highest *N*′ images are also noncircular, but again this is to a lesser extent than with N_2_. The V–H difference images show that there is a clear scattering-angle-dependent product rotational alignment, which again for high-*N*′ is dominated by alignment perpendicular to **k**. The fitted images are also in very good agreement with the data. The KA model is slightly less successful at predicting product polarization, particularly for the extreme forward scattered peak. One subtle, but distinct difference between the N_2_ and CO images is in the angular extent of the forward scattered peak. In the N_2_ images, this peak is essentially as sharp as it can be in our experiment, limited by the spreads of speeds in the molecular beams and the finite imaging resolution. In contrast, in the CO images the forward peak, while still very sharp, is noticeably wider at a low *N*′. This is reflected in the extracted DCSs, shown in Figure 7. For *N*′ = 5–8 from collisions with CO, the *E*_int_(1) forward peak falls to 10% of its initial value at ≈30°, while for the same product states with N_2_, it has reached the same level by ≈15°. Also in contrast to scattering from N_2_, there is no observed preference for sideways scattering for a high *N*′ in coincidence with rotational excitation of the collision partner, with the *E*_int_(1) scattering channel being the largest even for *N*′ = 9 and 10.

**Figure 8:**
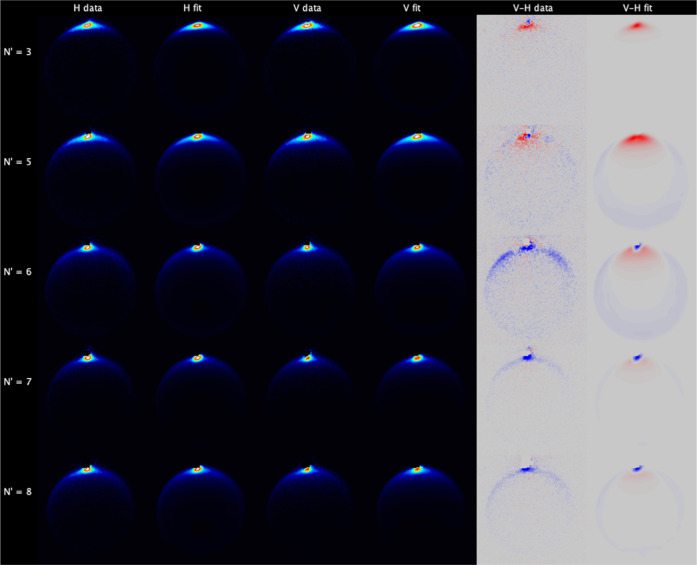
Experimental data and fits for NO(A) + O_2_. Left to right: H-polarization data; H-polarization fit; V-polarization data; V-polarization fit; V–H data subtraction; V–H fit subtraction. Top to bottom: *N*′ = 3, 5, 6, 7, and 8. The V–H subtraction color map ranges from blue (negative) to red (positive). The 6 experimental measurements for each *N*′ have been fitted independently, and the relevant images are summed for presentation here.

**Figure 9:**
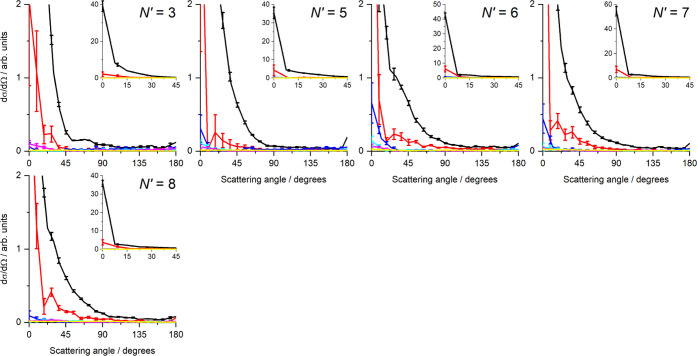
Differential cross sections (DCSs) for collisions with O_2_, as a function of O_2_ internal energy and for final NO *N′* = 3, 5–8. The total integral cross section for each final state has been normalized to unity. The main graphs span the full angular range (0–180°) but a reduced DCS range to enable comparison of DCSs for different O_2_ internal energies. The insets cover a limited angular range (0–45°) and the full DCS range, to enable comparison of the extreme forward scattering. Color scheme: *E*_int_(1) (black); *E*_int_(2) (red); *E*_int_(3) (blue); *E*_int_(4) (cyan); *E*_int_(5) (magenta); *E*_int_(6) (yellow). In each case, the error bars represent 1 standard error of the mean, resulting from the 6 independent experimental measurements of each *N*′.

Figure 8 shows the data and fit images for NO(A, *j* = 0.5) collisions with O_2_. These images again show a dominant, sharp, forward scattering peak, with only weak, low-intensity scattering away from this for all *N*′. The scattering that is visible in the forward hemisphere forms a narrow circular ring, with no evidence of distortion away from circularity, or of “in-filling,” that would indicate energy transfer to the O_2_. This is particularly clear for *N*′ = 8 when contrasted with the images recorded for collisions with N_2_, and to a lesser extent, CO. The V–H subtraction images indicate some rotational angular momentum alignment is present, with alignment parallel to **k** dominating for the forward scattered peak for *N*′ = 3 and 5, and alignment perpendicular to **k** dominating for all scattering angles for *N*′ = 6–8. The fit images are again in excellent agreement with the data, with no systematic deviations. The V–H fit images show broad agreement with the data, although for *N*′ = 6–8, the KA model predicts the opposite sign of difference in the ≈10–45° range. The DCSs shown in Figure 9 confirm the qualitative conclusions drawn from the inspection of the images. The scattering for all *N*′ peaks sharply at 0° and is almost exclusively in the forward hemisphere, and is very strongly dominated by the *E*_int_(1) channel, with only small contributions from the *E*_int_(2) channel. There is no contribution to sideways scattering from *E*_int_(≥1). The extreme forward scattering peak has the same appearance as that observed for N_2_, rather than CO, falling to below 10% of the peak by ≈15°, independent of *N*′.

**Figure 10:**
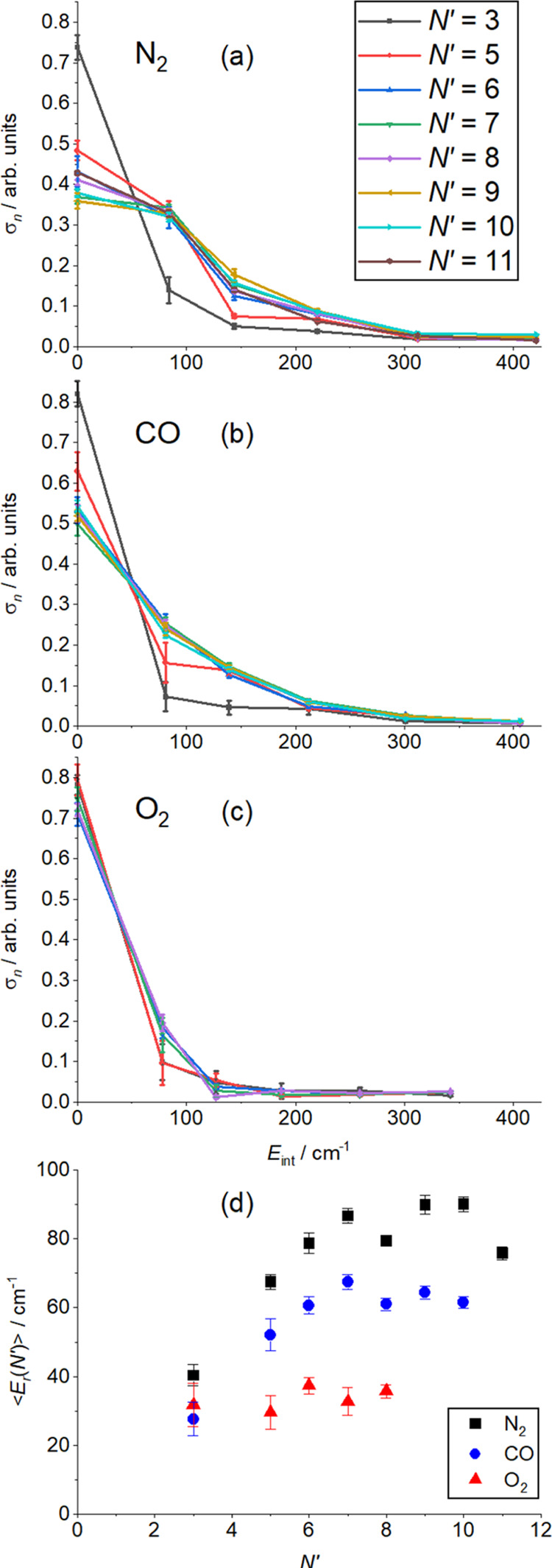
Integral cross sections, σ_*n*_(*N*′), as a function of internal energy in the collision partner, for final NO states, *N*′, for the three collision partners, (a) N_2_, (b) CO, and (c) O_2_. (d) Average internal energy of the collision partner, ⟨*E*_r_(*N*′)⟩, as a function of NO rotational level *N*′ for the three collision partners, N_2_ (black squares), CO (blue circles), and O_2_ (red triangles). In each case, the error bars represent one standard error.

The overall propensity for energy transfer to the collision partner can be described by the integral cross sections as a function of *E*_int_(*n*), σ_*n*_(*N*′). The DCSs extracted in the fitting procedure for each *N*′ and presented in Figures 5, 7, and 9 can be integrated over scattering angles dΩ to yield σ_*n*_(*N*′). The results are presented in Figure 10, where the sum of σ_*n*_(*N*′) over *n* for each *N*′ has been normalized to unity, and therefore no information is provided concerning the relative probabilities of scattering into different *N*′. Also included in Figure 10 is average internal energy ⟨*E*_r_(*N*′)⟩ as a function of *N*′ for each collider, derived from the relevant weighted sums of the σ_*n*_(*N*′). Figure 10 clearly shows that the transfer to internal energy in the collision partner is in the order N_2_ > CO > O_2_, for all measured *N*′. For both N_2_ and CO, there is a positive correlation between *N′* and *j*′ for *N*′ ≤ 7, followed by a relatively constant ⟨*E*_r_(*N*′)⟩ for *N*′ ≥ 8. There is very little product internal energy in the O_2_, where even for *N*′ = 8, more than 90% of the scattering is into *E*_int_(1) and *E*_int_(2), and ⟨*E*_r_(*N*′)⟩ is independent of *N*′. A reasonable question is thus whether there is any evidence for rotational excitation of the O_2_? In the test of the peeling algorithm on the NO(A) + Ne data, 99.2% of the total cross section was determined to be in *E*_int_(1) and *E*_int_(2), implying that even including the possibility of crosstalk between the *E*_int_(*n*) it is unlikely that the observed ≈10% scattered into *E*_int_(≥3) is a fitting artifact, and hence that there is some, albeit very limited, rotational excitation of the O_2_.

## Discussion

We first consider the results of NO(A) + N_2_ scattering. The results that are obtained from the peeling analysis presented here are consistent with those from the more limited fitting in our previous report on this system.^42^ There has been both theoretical and relevant experimental work on this system reported since our earlier study however, which we now consider. Petit and co-workers have performed ab initio calculations, with the primary intent of mapping and understanding the quenching mechanisms.^46^ They did locate a conical intersection leading to quenching of NO(A), but that lies behind a significant (0.404 eV, 3260 cm^–1^) barrier, consistent with the observed collision-energy dependence of the quenching cross section.^43^ This conical intersection will not be accessible at our experimental collision energy (790 cm^–1^), and we therefore expect the scattering of NO(A) + N_2_ here to only include A-state RET. Although there is no full van der Waals (vdW) potential available for this system, it has been well-established that the minimum is in a linear ON-N_2_ geometry, with a most recently reported well depth of −335 cm^–1^.^46,49^ It is therefore perhaps unsurprising that we see such strong forward scattering, as our extensive previous experiment and quantum scattering calculations on NO(A) + Rg RET has demonstrated that even relatively modest-depth attractive wells (e.g., −93 cm^–1^ for NO(A)-Ar)^50^ that are localized at the N-end of NO are efficient at inducing moderate Δ*N*′ with strong forward scattering.^28,30,31^ Indeed, the similarity of the forward scattering peaks in NO(A) + N_2_ to those observed in NO(A) + Ar is perhaps an indication that the NO(A)-N_2_ minimum is, like that in NO(A)-Ar, relatively tightly localized; the ON-Ar minimum ranges from approximately 0 to 40°.

Forward scattering with rotational excitation of the collision partner has recently been reported in the NO(X) + CO system, in a new, generally applicable, inelastic scattering mechanism that has been named “Hard-Collision Glory Scattering” (HCGS).^36,37^ There is no definitive signature of this mechanism in our DCSs. At the highest *N*′, the forward scattering is coincident with the lowest *j*′, with sideways scattering dominating for the higher *j*′, whereas the HCGS mechanism would result in forward scattering being preferred for the higher *j*′ coincident products. The contribution of the HCGS mechanism depends on the ratios of the well depth to collision energy, and inelastic energy transfer to collision energy, with a deep well and high inelastic energy transfer providing the preferred conditions. At 800 cm^–1^ collision energy, even for the *N*′ = 11 products, the *E*_int_(2) and *E*_int_(3) channels for which we see significant scattering cross section lie outside the energy ranges for which the HCGS mechanism is expected to be significant, consistent with their maxima lying to larger (sideways) scattering angles. The sideways and backward scattering that is observed for the higher-*N*′ NO states is of course consistent with low-impact-parameter collisions that sample the repulsive wall, giving rise to “rotational rainbow” scattering. In a crossed-beam experiment all impact parameters are of course necessarily sampled, and the signatures of low-impact-parameter collisions, namely, sideways and backward scattering correlated with rotational excitation, must generally appear in the data.

The N_2_ average rotational energy, ⟨*E*_r_(*N*′)⟩, shown in Figure 10 is clearly positively correlated with *N*′, and in the range 80–90 cm^–1^ for *N*′ ≥ 7 corresponds to an average *j*′ = 6. This is broadly consistent with similar anisotropy in the PES for NO relative to N_2_ and vice-versa. Recent experiments probing the NO(A, *N*′) produced by the photodissociation of NO(A)-N_2_ van der Waals complexes have observed considerably higher N_2_ than NO rotational energy, in particular at lower excess energy.^51−53^ However, these experiments necessarily start from a constrained initial geometry dictated by the NO(X)-N_2_ collision complex (X-shaped) and its Franck-Condon overlap with the NO(A)-N_2_ complex (Linear ON-N_2_). Hence, although they clearly show that substantial anisotropy exists in the NO(A)-N_2_ PES that can lead to N_2_ rotational excitation, they sample that PES differently, and are not directly comparable to our experiments. Finally, the observed rotational alignment correlations are also consistent with this picture of the scattering. At high-*N*′ and for sideways and backward scattering, good agreement is seen with the KA model, across the range of *E*_int_(*n*), as expected for the more rigid, impulsive, scattering seen in low-impact-parameter collisions that sample the repulsive wall.^3^ The agreement of the KA model with the data is less good for the forward-scattered peak, as has previously been observed in NO(X)/(A) scattering where attractive forces are dominant.^54^

We now turn to the NO(A) + CO system. The study by Petit and co-workers on the NO(A) + N_2_ system also includes similar calculations on the NO(A) + CO system, again with the primary aim of locating conical intersections that might be responsible for the observed, moderate, quenching cross sections.^43,46^ They located a barrierless, short-range, conical intersection in this system, which is accessible from long range. Investigation of the longer-range potential found a van der Waals minimum for the O–N–C–O geometry with a well depth of −460 cm^–1^, with substantial anisotropy with respect to both NO and CO rotation at this internuclear separation (*R* = 3 Å). The O–N–C–O minimum is notable for its width with respect to the ONC angle, extending from θ_ONC_ = 100 to 180°.

**Figure 11:**
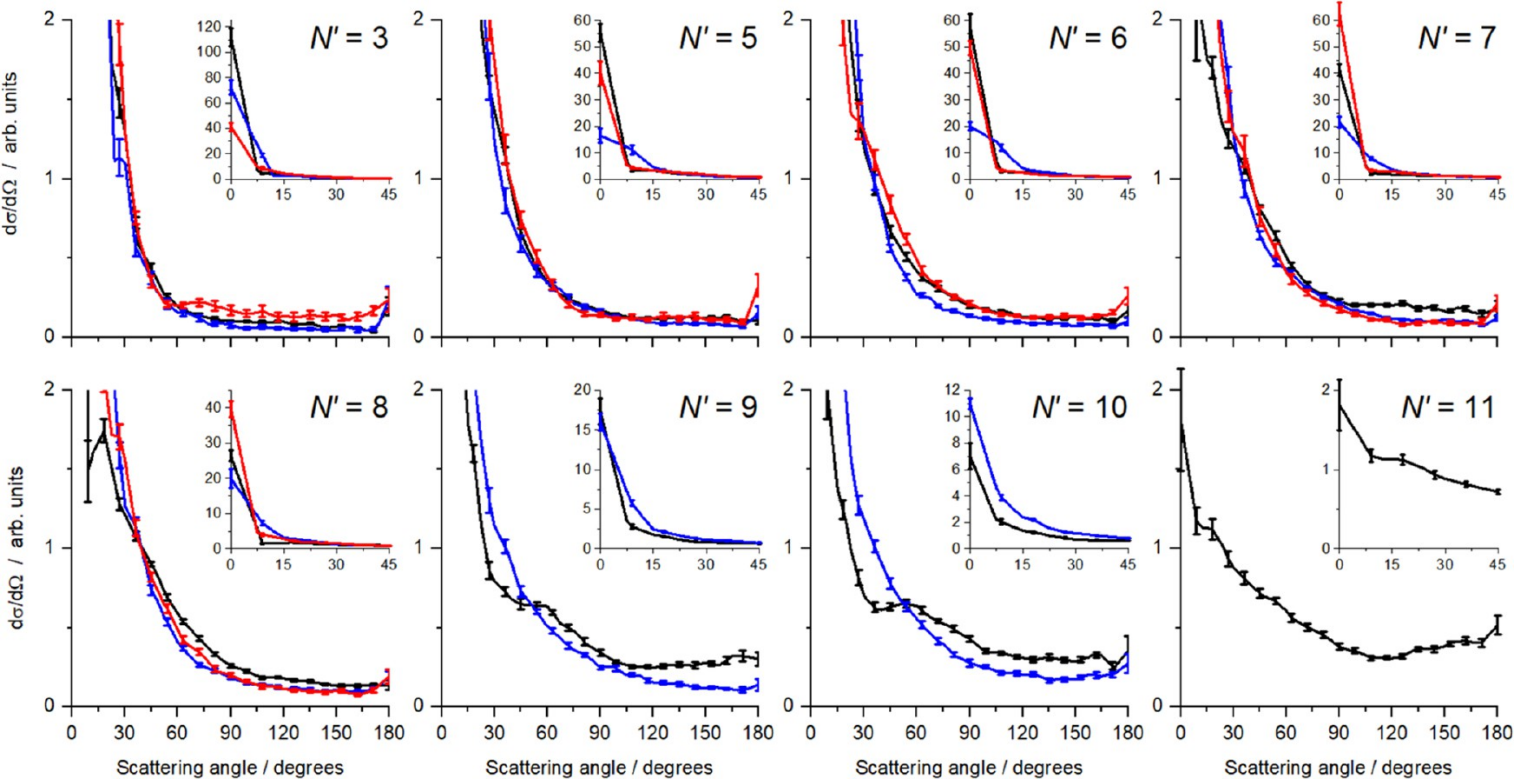
Total differential cross sections, summed over all collider internal energies *E*_int_(*n*), as a function of NO final state, *N*′, for the three collision partners, N_2_ (black), CO (blue), and O_2_ (red). The main graphs span the full angular range (0–180°) but a reduced DCS range to enable comparison of DCSs for sideways and backward scattering angles. The insets cover a limited angular range (0–45°) and the full DCS range, to enable comparison of the extreme forward scattering. For each collider and *N*′, the integral cross section is separately normalized to unity, and the error bars represent 1 standard error.

Experimentally, we observe strong forward scattering in NO(A) + CO, with overall less sideways and backward scattering than is observed with N_2_. This is particularly clear in the total DCSs, summed over all *E*_int_(*n*), which are shown in Figure 11 for all three collision partners. It is also apparent that the forward scattering peak is broader than that for N_2_. These observations are consistent with an overall more attractive PES for NO(A)-CO than for NO(A)-N_2_, supported by the available information on the vdW PESs. That the collisions are more strongly mediated by attractive than repulsive interactions is also supported by the V–H difference images, where the agreement of the KA model is poorer for NO(A) + CO than it was for NO(A) + N_2_. The same energetic arguments presented above for NO(A) + N_2_ discounting the HCGS mechanism as a source of forward scattered NO in coincidence with rotationally excited N_2_ apply to NO(A) + CO as well. Similarly, there is no evidence for scattering with correlated high rotational energies in both fragments, as observed in the CO + CO system.^41^ The broader angular ranges of this forward scattering compared to that observed for NO(A) + N_2_ are perhaps a reflection of the wide angular range of the attractive well, rather than it being tightly focused around the linear ON–CO geometry.

There is overall lower rotational excitation in the CO than in the N_2_, although the relative dependence on *N*′ is very similar. This is surprising; both the available PES information and the presence of dipole–dipole interactions in NO(A)-CO which are not present in NO(A)-N_2_ suggest that overall CO should be expected to experience greater anisotropy than N_2_ in a collision with NO(A). All other things being equal, this should lead to greater rotational excitation of CO than N_2_. Is there any evidence for the presence of the quenching channel in these RET measurements? The conical intersection located by Petit and co-workers is at a short range, so should be primarily accessed by lower-impact-parameter collisions. This could be a factor in the lower rotational excitation of the CO, and the relative lack of sideways and backward scattering. However, there are no clear dynamical signatures that unambiguously indicate the existence of the quenching channel.

Finally, we turn to the NO(A) + O_2_ system. The observed scattering dynamics for NO(A) + O_2_ are substantially different from those for CO and N_2_. Very little sideways or backward scattering is observed, with scattering dominated by a sharp forward peak. The scattering is dominated by the elastic, *E*_int_(1), channel, with very little rotational excitation of the O_2_ resulting in ⟨*E*_r_(*N*′)⟩ ≈ 30 cm^–1^, regardless of *N*′. These results are very surprising, particularly in light of the vdW PES that we recently published for NO(A) + O_2_.^47^ This PES has an N-end minimum, depth −95 cm^–1^, tightly focused around the linear ON–OO geometry, very similar to those also observed in the NO(A)-Ar PES.^50^ This is fully consistent with the observed sharp forward scattering peak. However, at the range (*R* = 4.3 Å) of the minimum, the PES also displays significant anisotropy as a function of the orientation of both the NO and O_2_. For example, in the “hammer” geometry, with O pointing at the mid-bond of NO, *V*(*R* = 4.3 Å) ≈ +200 cm^–1^. On the very similar NO(A)-Rg PES, such anisotropy would lead to rotational excitation of the NO from scattering on the repulsive wall of the PES, characterized by the sideways and backward angles of rotational rainbow scattering. The anisotropies predicted are also consistent with expected rotational excitation of the O_2_, via essentially sideways and backward scattering in which both NO and O_2_ undergo rotational excitation. But no such scattering is observed in the experimental results. We emphasize again that in a crossed-beam experiment such as this, all impact parameters and relative geometries are necessarily sampled, and therefore the scattering resulting from them should appear in the observable final states.

The obvious possibility is that the missing scattering is the result of quenching. A typical total inelastic scattering cross section for a diatom–diatom system, consistent with the vdW PES, would be ≈75 Å^2^. We would therefore expect ≈1/3 of collisions to result in quenching of NO(A), from the literature quenching cross section of ≈25 Å^2^.^43^ We have recently identified quenching pathways through conical intersections on both doublet and quartet PESs of NO(A)-O_2_.^48^ On the doublet PES, a short range (*R* = 2.5 Å) barrierless intersection is found at a well-defined nonlinear ON-O_2_. Additional intersections exist at longer ranges, similar to the range of the vdW minimum, on both the doublet and quartet PESs. However, these are away from the linear ON-O_2_ geometry that characterizes that vdW minimum. The locations of these intersections are consistent with the experimental measurements of Few et al. and Blackshaw et al., which clearly show that the NO(X) is formed both vibrationally and rotationally excited and that the O_2_ is also formed with significant internal excitation, either vibrational or electronic (*c*^1^Σ_*u*_^–^).^44,45^ An interpretation of our results is therefore that the absence of sideways and backward scattering, or of significant rotational excitation of the O_2_, is the result of collisions that preferentially undergo quenching because they sample geometries away from linear and at shorter range resulting from lower-impact parameters. We are left with the unquenched NO(A) + O_2_ products that underwent high-impact-parameter collisions and largely sampled the linear geometry vdW minimum, consequently undergoing glory scattering producing forward-scattered products with low rotational excitation in both fragments.

## Conclusions

We have measured the rotational-state-correlated differential cross sections for inelastic scattering of NO(A, *j* = 0.5) with N_2_, CO, and O_2_, at collision energies close to 800 cm^–1^. The DCSs for all product NO *N*′ rotational levels are forward peaked, but the extent of sideways and backward scattering is strongly dependent on the collision partner, in the order N_2_ > CO > O_2_. This same order is observed for the extent of the rotational excitation of the collider, with little or no rotational excitation observed for O_2_. The observed scattering dynamics for collisions with N_2_, and to a lesser extent, CO, are explicable in terms of known details of the vdW PESs for the NO(A)-N_2_ and NO(A)-CO systems.^46,47^ The dynamics observed for collisions with O_2_ are consistent with only high-impact-parameter glory scattering, while there is an absence of scattering arising from the lower-impact parameter, repulsive wall collisions. Considering the literature NO(A) electronic quenching cross sections, and recent electronic structure calculations for all three systems, we interpret this as the result of quenching removing NO(A) that undergoes lower-impact-parameter collisions with O_2_.^43,46−48^ The dominance of forward-scattered, near-elastic, collisions is thus a signature of the presence of the conical intersections that lead to NO(A) quenching in collisions with O_2_.

## Data Availability

The data underlying this study are openly available in the Heriot-Watt University archive at https://doi.org/10.17861/d9cb0f39-74e4-4a83-b2f6-9509a37b2b4c
